# Systemic Inflammation, Tumor Isotopic Signatures, and Prognosis in Oral Squamous Cell Carcinoma: Exploratory Integration of Blood- and Tissue-Derived Biomarkers—An Exploratory Retrospective Secondary Analysis

**DOI:** 10.3390/jcm15135278

**Published:** 2026-07-06

**Authors:** Katarzyna Bogusiak, Piotr Paneth, Marcin Majchrzak, Marcin Kozakiewicz, Józef Kobos

**Affiliations:** 1 Department of Maxillofacial Surgery, Medical University of Lodz, 251 Pomorska, 92-209 Lodz, Poland; marcin.majchrzak@csk.umed.pl (M.M.);; 2 Institute of Applied Radiation Chemistry, Lodz University of Technology, 116 Żeromskiego, 90-924 Lodz, Poland; piotr.paneth@p.lodz.pl; 3 Department of Histology and Embryology, Medical University of Lodz, 251 Pomorska, 92-209 Lodz, Poland; jozef.kobos@umed.lodz.pl; 4 Department of Pathology, Medical University of Lodz, 251 Pomorska, 92-209 Lodz, Poland

**Keywords:** squamous cell carcinoma of head and neck, mass spectrometry, neutrophils, lymphocytes, carbon isotopes, nitrogen isotopes, neoplasms, metabolism, disease-free survival, retrospective studies

## Abstract

**Background/Objectives**: Oral squamous cell carcinoma (OSCC) remains clinically heterogeneous, and prognosis is not always fully explained by conventional clinicopathological parameters. Systemic inflammation and tumor metabolic alterations may provide complementary information on tumor biology. This study aimed to assess associations between preoperative inflammatory markers, isotope ratio mass spectrometry (IRMS)-derived tumor signatures, clinicopathological features, and survival outcomes in OSCC. **Methods**: This exploratory retrospective secondary analysis included 50 consecutive patients with surgically treated, histologically confirmed OSCC. Preoperative blood-based markers, including neutrophil-to-lymphocyte ratio (NLR), platelet-to-lymphocyte ratio (PLR), systemic immune-inflammation index (SII), white blood cell count, lymphocyte count, and C-reactive protein, were retrieved from routine laboratory tests. Matched tumor, surgical margin, and healthy oral mucosa samples were analyzed by IRMS for δ^13^C, δ^15^N, carbon and nitrogen content, and [N]/[C] ratio. Associations with clinicopathological variables, nodal status, overall survival (OS), and disease-free survival (DFS) were evaluated using non-parametric tests, Spearman correlations, and Cox regression models. **Results**: Tumor tissue showed a consistent isotope and elemental phenotype compared with healthy mucosa, including higher nitrogen content, lower carbon content, increased [N]/[C] ratio, lower δ^15^N, and less negative δ^13^C values. NLR, PLR, SII, and CRP were not robustly associated with standard clinicopathological features after correction for multiple testing. Correlations between inflammatory and isotope-derived parameters were modest. Higher NLR was associated with worse OS and DFS and remained significant after adjustment for pathologic nodal status. Less negative tumor δ^13^C showed a potential adverse prognostic signal. **Conclusions**: Systemic inflammatory markers and IRMS-derived tumor signatures appear to reflect partly distinct biological domains in OSCC. NLR may provide accessible prognostic information, while tumor δ^13^C warrants further validation as a metabolic biomarker.

## 1. Introduction

Oral squamous cell carcinoma (OSCC) remains a major global health burden, accounting for the vast majority of malignancies arising in the oral cavity [1]. Despite advances in surgical techniques, radiotherapy, and systemic treatments, the prognosis of OSCC patients remains highly variable and, in many cases, difficult to predict based only on conventional clinicopathological parameters. Even within similar TNM stages, substantial heterogeneity in clinical outcomes is observed, highlighting the need for additional, biologically informative prognostic markers [2].

In recent years, increasing attention has been directed toward systemic inflammatory markers derived from routine blood tests as potential predictors of cancer behavior. Ratios such as the neutrophil-to-lymphocyte ratio (NLR), platelet-to-lymphocyte ratio (PLR), and composite indices such as the systemic immune-inflammation index (SII) reflect the balance between pro-tumor inflammatory processes and anti-tumor immune responses [3,4,5]. Elevated values of these markers, alongside increased C-reactive protein (CRP), have been associated with tumor progression, lymph node metastasis, and poorer survival outcomes in multiple malignancies, including head and neck cancers [3,5,6,7,8]. Importantly, these biomarkers are inexpensive, easily calculated, and readily available in everyday clinical practice, making them attractive candidates for risk stratification [7,9]. However, as non-tumor-specific indicators of systemic inflammation, they may be influenced by intercurrent infection, comorbidities, medications, smoking status, and nutritional condition.

At the same time, cancer metabolism has emerged as a key hallmark of tumor biology [10]. Neoplastic cells exhibit profound metabolic reprogramming, which can be reflected in the isotopic composition of tumor tissues [10]. Stable isotope ratio analysis, including measurements of carbon (δ^13^C) and nitrogen (δ^15^N) isotopic signatures, as well as elemental composition ([C], [N], and their ratios), provides insight into an integrated tissue-level signature reflecting metabolic pathways active within the tumor microenvironment [11,12]. Such approaches offer a unique perspective on tumor biology that is complementary to traditional histopathological and molecular assessments [11].

Although systemic inflammation and tumor metabolism represent distinct biological domains, there is growing evidence that they are closely interconnected. Inflammatory processes can influence metabolic pathways within the tumor microenvironment, while metabolic alterations may, in turn, modulate immune responses [13]. However, the relationship between easily accessible systemic inflammatory markers and the isotopic profile of tumor tissues remains poorly understood, particularly in OSCC [11,14].

Furthermore, it is unclear whether combining information from these two domains—systemic inflammation and tumor metabolic signatures—can improve prognostic stratification beyond what is achievable with each approach alone [14]. Integrating blood-based biomarkers with tissue-derived metabolic data may provide a more comprehensive representation of tumor behavior and host–tumor interactions [13].

Therefore, the aim of this study was to evaluate the association between preoperative systemic inflammatory markers and clinicopathological features, as well as survival outcomes, in patients with OSCC. Additionally, we sought to investigate the relationship between inflammatory indices and isotopic parameters of tumor and margin tissues. Finally, we explored whether a combined model incorporating both inflammatory and isotopic biomarkers could enhance risk stratification in this patient population.

## 2. Materials and Methods

### 2.1. Study Design

This study was designed as a retrospective secondary analysis of prospectively collected data. Patients were recruited between 25 June 2018 and 29 July 2020. Overall, 50 consecutive patients fulfilling the criteria of this analysis were enrolled. Spectrometric measurements and isotopic abundance of carbon and nitrogen in tissue samples were obtained prospectively as a part of a primary research protocol involving patients with histologically confirmed oral squamous cell carcinoma (OSCC). The clinicopathological data was collected, including demographic characteristics, primary tumor site and staging, histopathological features and follow-up information.

Tumors were classified according to the 8th edition of the AJCC/UICC TNM staging system [15]. Nodal status was analyzed both as a categorical variable and as a binary variable (pN0 vs. pN+).

For the present analysis, routinely collected inflammatory markers were retrieved retrospectively from electronic medical records. These markers were not a part of the original study objectives and were analyzed post hoc to explore their potential associations with spectrometric parameters.

No additional interventions or patient contact were required for this secondary analysis.

This study was reported in accordance with the Strengthening the Reporting of Observational Studies in Epidemiology (STROBE) guideline for observational studies (Appendix A, Table A1). Generative artificial intelligence tools were used during manuscript preparation only to support language editing and to assist in searching for publicly available information (OpenAI ChatGPT, GPT-5.5 Thinking, San Francisco, CA, USA).

### 2.2. Patient Cohort

Patients were recruited consecutively from a single tertiary referral center.

Inclusion criteria comprised:

- Primary, locally advanced OSCC (confirmed by histopathological examination);
- Eligibility for surgical treatment with curative intent;
- Availability of tumor, surgical margin tissue and reference healthy oral mucous membrane sample for paired histopathological and spectrometric analysis.

Exclusion criteria comprised:

- Prior radiotherapy or chemotherapy of the head and neck region;
- Malnutrition (BMI ˂ 18.5) or diabetes mellitus;
- Incomplete clinical or laboratory data.

All patients underwent mandatory pre-operative dental evaluations as a part of the standard oncological qualification protocol. Active periodontal inflammation was excluded in every case, and no patient required periodontal interventions prior to surgery. No participant followed specific dietary restrictions or exclusionary regimens. None of the study group suffered from active systemic inflammatory or autoimmune disease nor was on chronic corticosteroid or immunosuppressive therapy.

Diabetes mellitus was excluded because chronic metabolic dysregulation and systemic inflammation associated with diabetes may substantially affect inflammatory biomarkers and potentially influence tissue metabolic signatures [16,17].

All patients underwent standard therapeutic procedures (tumor resection, neck dissection) and adjuvant treatment according to current clinical guidelines by the National Comprehensive Cancer Network (NCCN) [18].

Patients underwent follow-up visits scheduled at three-month intervals. Disease recurrences (local, regional/nodal or distant) were evaluated based on clinical examination, imaging studies and histopathological confirmation when indicated.

The primary endpoints were overall survival (OS) and disease-free survival (DFS). Survival times were calculated in weeks from the date of surgery to the event of interest or last follow-up. OS was defined as time to death from any cause, while DFS was defined as time to the first documented recurrence or death.

This study was approved by the Bioethics Committee (RNN/185/18/KE).

### 2.3. Blood-Based Inflammatory Markers

Preoperative laboratory parameters were retrieved retrospectively from blood samples obtained at a standardized time point—1 day before surgery as part of routine clinical care. The following parameters were extracted: white blood cell count (WBC), neutrophil count, lymphocyte count, platelet count, and C-reactive protein (CRP).

Based on these values, the following inflammatory indices were calculated:

- Neutrophil-to-lymphocyte ratio (NLR);
- Platelet-to-lymphocyte ratio (PLR);
- Systemic immune-inflammation index (SII = platelet × neutrophil/lymphocyte).

All laboratory tests were performed in the hospital’s accredited diagnostic laboratory using standardized methods. CRP was analyzed both as a continuous variable and as a binary variable using a predefined threshold (CRP_high). For the main analyses, all inflammatory markers were treated as continuous variables (without applying arbitrary cut-off values) to avoid information loss associated with categorization.

### 2.4. Sample Collection and Preparation

Four tumor tissues, 4 from surgical margins and two corresponding healthy tissue samples were collected intraoperatively immediately after resection from each patient. The collected sets of specimens were handled according to a standardized protocol for IRMS analysis and histopathological assessment. First sets of matched samples (2 from tumor, 2 from margins, 1 from healthy oral mucosa) were immersed in formalin, embedded in paraffin and evaluated by an experienced pathologist (J.K.). Samples derived from surgical margins and normal-appearing mucosa were histologically reviewed to ensure that no pathological changes—such as tumor infiltration, dysplasia or other premalignant alterations—were present. Additionally, the entire postoperative tumor and lymph node specimens underwent routine histopathological examination.

The second sets of corresponding tissue samples were frozen immediately after collection at −70 °C. Prior to spectrometric analysis, samples were lyophilized using a Christ Delta 1-24 Lsc lyophilizer (GmbH, Osterode am Harz, Germany), homogenized and weighed into tin capsules (3 ± 1 mg). On average, 3 subsamples were analyzed per one tissue section.

### 2.5. Isotope Ratio Mass Spectrometry (IRMS)

Stable isotope analysis was performed using a Sercon (Sercon Ltd., Crewe, Cheshire, UK) SL20-22 continuous-flow isotope ratio mass spectrometer coupled with a Sercon SL elemental analyzer (Sercon Ltd., Crewe, Cheshire, UK) to simultaneously quantify carbon and nitrogen isotope ratios.

The following parameters were analyzed:

- δ^13^C and δ^15^N values (‰), expressed relative to international standards (PDB for carbon and AIR for nitrogen);
- Total carbon (C) and nitrogen (N) content (%);
- Nitrogen-to-carbon ratio ([N]/[C]).

In addition, derived variables were calculated to capture metabolic differences between tissues, including tumor–margin and tumor–healthy tissue gradients (ΔT–M and ΔT–HT). In accordance with the predefined analysis plan, the primary focus was placed on tumor and tumor–healthy tissue comparisons.

Calibration was performed using certified reference materials measured at regular intervals. Analytical precision was monitored through repeated measurements of internal laboratory standards.

### 2.6. Statistical Analysis

Continuous variables were summarized as medians with interquartile ranges (IQRs), whereas categorical variables were presented as counts and percentages. Given the limited sample size and the expected non-normal distribution of inflammatory and isotope-derived biomarkers, non-parametric methods were used for group comparisons. Paired comparisons of isotope and elemental parameters between tumor tissue, surgical margin, and healthy oral mucosa were performed using the Wilcoxon signed-rank test. Independent two-group comparisons, including analyses according to pathologic nodal status and other clinicopathological features, were performed using the Mann–Whitney U test, with rank-biserial correlation used as an effect-size measure where appropriate. Associations between continuous inflammatory markers and isotope-derived variables were assessed using Spearman’s rank correlation coefficient. To account for multiple testing, *p*-values from families of related analyses were adjusted using the Benjamini–Hochberg false discovery rate procedure. Overall survival and disease-free survival were analyzed using Cox proportional hazards regression. Continuous biomarkers were analyzed as continuous variables and scaled per interquartile range increase unless otherwise specified. Kaplan–Meier curves stratified by median biomarker values were used for visualization only, whereas statistical interpretation was based primarily on continuous Cox models. Multivariable Cox models were restricted to a limited number of clinically justified covariates because of the sample size and number of events. Collinearity between covariates was assessed using variance inflation factors. All analyses were considered exploratory, and two-sided *p*-values below 0.05 were regarded as statistically significant.

Given the relatively small sample size and the number of exploratory comparisons performed, the analyses were considered hypothesis-generating. The risk of type I statistical error and model overfitting cannot be excluded despite the use of false discovery rate correction and parsimonious multivariable modeling.

## 3. Results

### 3.1. Patient Cohort and Oncologic Outcomes

A total of 50 patients with oral squamous cell carcinoma were included in the study. The median age of the cohort was 68 years, with an interquartile range of 60.3 to 71 years, indicating that the study population consisted predominantly of older adults. Men constituted the majority of the cohort (32 male patients, 64.0% of the study group), whereas 18 patients were female, accounting for 36.0%. The selected clinicopathological features of the study cohort are summarized in Table 1.

Tumors were most frequently located in the lower gingiva, in 20 patients (representing 40.0% of the cohort) and floor of the mouth identified in 17 patients (34.0%). Most patients had advanced disease: 24 patients (48.0%) had pT4 tumors and 39 (78.0%) were classified as stage IV according to the 8th edition of the AJCC staging system. Pathologic nodal metastases were present in 29 patients (58.0%), and extranodal extension was identified in 23 patients (46.0%). Regarding histopathological grading, moderately differentiated tumors were the most common. Grade 2 tumors were identified in 31 patients, corresponding to 62.0% of the cohort. In this study, a substantial subset of the cohort demonstrated pathological features associated with aggressive tumor behavior, including extranodal extension (ENE), which was identified in 23 patients (46.0%), angioinvasion (present in 18 patients; 36.0%) and neuroinvasion (observed in 16 patients; 32.0%).

During follow-up, locoregional recurrence occurred in 17 patients (34.0%), distant metastases in 10 patients (20.0%), and death in 31 patients (62.0%). The median OS time was 208 weeks (IQR: 157–309.5), whereas the median DFS time was 181.5 weeks (IQR: 71–285.25).

### 3.2. Preoperative Systemic Inflammatory Profile

Preoperative inflammatory markers showed substantial inter-patient variability. The median NLR was 3.11 (IQR: 2.39–4.75), while the median PLR was 151.04 (IQR: 108.14–190.44) (Table 2). The median SII was 704.62 (IQR: 530.42–1317.19), showing substantial variability in the combined platelet-, neutrophil-, and lymphocyte-based inflammatory profile of the cohort. The median preoperative CRP concentration was 6.55 mg/L (IQR: 2.25–15.35). After dichotomization using a 5 mg/L cut-off, 30 patients (60.0%) had CRP levels above 5 mg/L, whereas 20 patients (40.0%) had CRP values of 5 mg/L or lower. These findings indicate that systemic inflammatory activation was common in the study population and support further analyses evaluating the prognostic and clinicopathological relevance of these markers.

### 3.3. Matched Tumor, Margin, and Healthy Tissue Isotope Profiles

**Figure 1:**
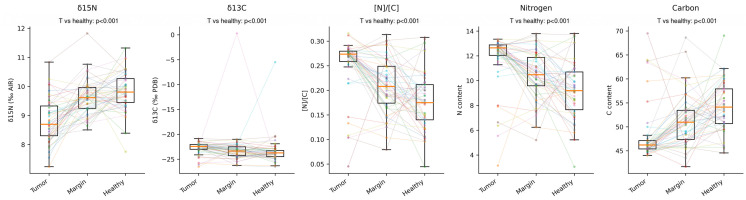
Matched tissue isotope and elemental profiles. Boxplots and paired patient-level trajectories show tumor, margin, and healthy mucosa values for δ^15^N, δ^13^C, [N]/[C], nitrogen content, and carbon content. The figure highlights robust tumor–healthy gradients, supporting a tumor-specific isotope/elemental phenotype.

Matched tissue comparisons revealed a consistent isotope and elemental gradient across tumor tissue, surgical margin, and healthy oral mucosa (Table 3 and Figure 1). Tumor tissue showed higher nitrogen content than both margin tissue and healthy mucosa, with a median paired tumor–healthy difference of 3.19 (IQR: 1.64–4.22; Wilcoxon *p* < 0.001; FDR *q* < 0.001). In contrast, tumor carbon content was lower than in healthy mucosa, with a median paired tumor–healthy difference of −6.49 (IQR: −10.83 to −2.66; *p* < 0.001; *q* < 0.001). The [N]/[C] ratio was also higher in tumor tissue than in healthy mucosa (median paired difference: 0.09, IQR: 0.04–0.12; *p* < 0.001; *q* < 0.001). Isotopically, tumor tissue had lower δ^15^N than healthy mucosa (median paired difference: −1.04‰, IQR: −1.52 to −0.52; *p* < 0.001; *q* < 0.001) and less negative δ^13^C than healthy mucosa (median paired difference: 1.16‰, IQR: −0.03–1.84; *p* < 0.001; *q* < 0.001). These findings support the presence of a reproducible tumor-specific metabolic/isotopic phenotype in OSCC.

### 3.4. Associations Between Inflammatory Markers and Clinicopathological Features

In univariable analyses, preoperative inflammatory markers were not significantly associated with conventional clinicopathological indicators of tumor aggressiveness after correction for multiple testing (Table 4). Specifically, NLR, PLR, SII, and CRP showed no significant relationships with pT category, pN category, stage, depth of invasion category, extranodal extension, angioinvasion, or neuroinvasion in the primary non-parametric and logistic regression analyses. Thus, systemic inflammatory status did not appear to reflect standard histopathological measures of aggressiveness in a robust manner.

### 3.5. Biomarkers and Pathologic Nodal Disease

**Figure 2:**
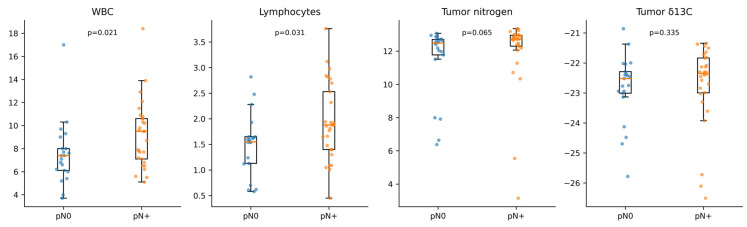
Selected biomarker distributions according to pathologic nodal status. WBC and lymphocyte counts were higher in pN+ patients, while tumor nitrogen content showed a trend toward higher values. These findings are exploratory because FDR-corrected significance was not retained.

When biomarkers were compared according to pathologic nodal status, pN+ patients had higher WBCs than pN0 patients (median 9.50 vs. 7.40; Mann–Whitney *p* = 0.021) and higher lymphocyte counts (median 1.88 vs. 1.55; *p* = 0.031). Tumor nitrogen content showed a trend toward higher values in pN+ disease (median 12.72 vs. 12.49; *p* = 0.065). NLR, PLR, SII, and CRP did not differ significantly between pN0 and pN+ groups. The number of metastatic lymph nodes correlated weakly with WBC count (Spearman ρ = 0.289, *p* = 0.042). These observations were shown in Figure 2 and Table 5. After FDR correction, none of the between-group biomarker associations with nodal status remained statistically significant; therefore, these findings should be interpreted as exploratory signals rather than definitive nodal biomarkers.

### 3.6. Correlations Between Inflammatory Markers and IRMS Parameters

**Figure 3:**
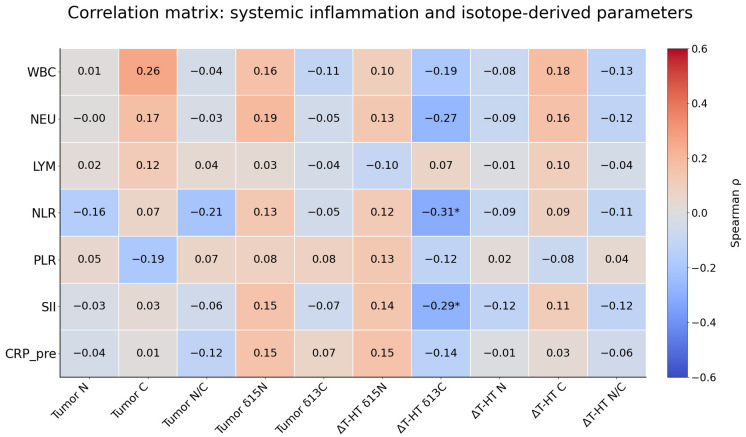
Spearman correlation matrix between systemic inflammatory markers and selected isotope-derived parameters. Correlations were generally modest, suggesting that systemic inflammation and tissue isotope signatures provide partly distinct biological information. Values represent Spearman’s correlation coefficients (ρ). * *p* < 0.05.

Correlation analysis demonstrated only modest associations between systemic inflammatory indices and tissue isotope-derived parameters (Figure 3). The strongest nominal correlations were observed between NLR and the tumor–healthy δ^13^C gradient (Spearman ρ = −0.306, *p* = 0.031) and between SII and the tumor–healthy δ^13^C gradient (ρ = −0.295, *p* = 0.038). However, these correlations did not remain significant after FDR correction. In the pN+ subgroup, the observed associations became more pronounced. Overall, the data suggest that blood-based inflammatory indices and tissue isotope signatures reflect partially overlapping but not redundant biological domains.

### 3.7. Survival Analyses

**Figure 4:**
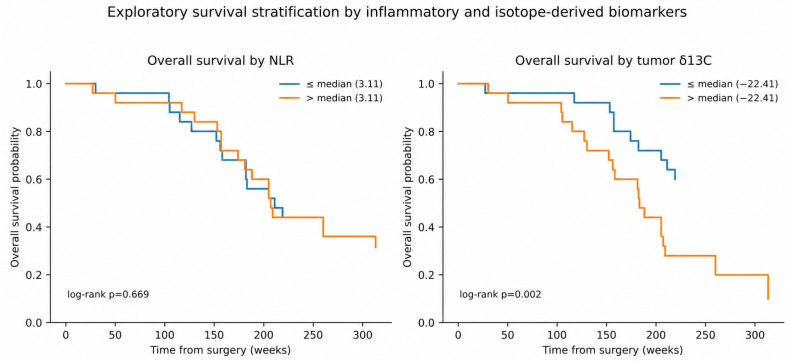
Exploratory Kaplan–Meier curves for OS stratified by median NLR and median tumor δ^13^C. These plots are intended for visualization; continuous Cox models should be used for statistical interpretation.

Patients who died during follow-up had less negative tumor δ^13^C values than survivors (median −22.30‰ vs. −22.94‰; Mann–Whitney *p* = 0.007). A similar pattern was observed for DFS events, with less negative tumor δ^13^C values among patients experiencing recurrence or death (median −22.30‰ vs. −22.99‰; *p* = 0.005). Healthy-tissue δ^13^C also differed according to OS event status (*p* = 0.029). In univariable Cox regression, higher NLR was associated with worse OS (HR per IQR increase: 1.14; 95% CI: 1.01–1.30; *p* = 0.041) and worse DFS (HR per IQR increase: 1.16; 95% CI: 1.00–1.33; *p* = 0.047). Pathologic nodal positivity was associated with worse OS (HR: 2.21; 95% CI: 1.03–4.72; *p* = 0.041), while its association with DFS did not reach statistical significance. Tumor δ^13^C showed a borderline association with both OS and DFS in Cox models, supporting its potential prognostic relevance but requiring validation in a larger cohort. Detailed data is presented in Table 6 and Figure 4. The lack of significance in the median-split analysis does not invalidate the continuous model, but demonstrates the limited usefulness of simple dichotomization of the NLR.

### 3.8. Multivariable Survival Models

**Figure 5:**
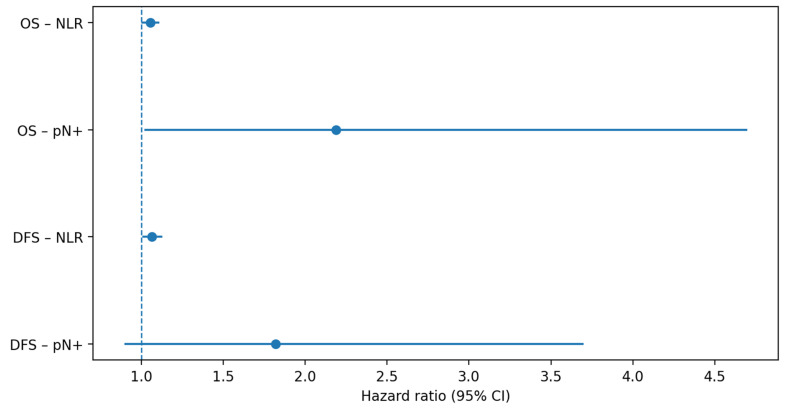
Primary multivariable Cox model (NLR + pN).

In the primary multivariable Cox model including NLR and pN status, NLR remained an independent predictor of both overall survival and disease-free survival (Figure 5). For overall survival, NLR retained statistical significance (HR 1.054, 95% CI 1.001–1.110, *p* = 0.048), while pN positivity also remained independently associated with worse overall survival (HR 2.188, 95% CI 1.019–4.699, *p* = 0.045). For disease-free survival, NLR remained independently associated with worse outcome (HR 1.066, 95% CI 1.006–1.130, *p* = 0.031), whereas pN status showed only a trend (HR 1.821, 95% CI 0.896–3.701, *p* = 0.098).

A second exploratory model including NLR, pN status, and SII showed significant associations of NLR (HR 1.333, 95% CI 1.013–1.754, *p* = 0.040) and pN status (HR 2.813, 95% CI 1.231–6.431, *p* = 0.014) with overall survival. However, SII itself was not significant, and variance inflation factor analysis demonstrated severe multicollinearity between NLR and SII (Supplementary Figure S1), indicating that this model should be interpreted with caution and regarded as exploratory only.

## 4. Discussion

In this study, we investigated the relationship between systemic inflammatory markers and tumor metabolic characteristics assessed by isotope ratio mass spectrometry (IRMS), as well as their prognostic significance in patients with oral squamous cell carcinoma (OSCC).

The main findings can be summarized as follows: (1) systemic inflammatory markers showed no significant associations with classical clinicopathological indicators of tumor aggressiveness, (2) selected correlations between inflammatory markers and metabolic tumor–healthy tissue gradients were observed, particularly in patients with nodal metastases, and (3) neutrophil-to-lymphocyte ratio (NLR) emerged as an independent prognostic factor for both overall survival (OS) and disease-free survival (DFS).

One of the most consistent findings of this study was the presence of a tumor-specific isotope and elemental phenotype. Compared with matched healthy oral mucosa, OSCC tissue showed higher nitrogen content, lower carbon content, an increased [N]/[C] ratio, lower δ^15^N, and less negative δ^13^C values. These paired differences remained significant after false discovery rate correction, indicating that IRMS-derived parameters capture robust biochemical differences between malignant and non-malignant oral tissues. Importantly, this tissue-level signal was stronger and more reproducible than the associations observed between systemic inflammatory markers and conventional clinicopathological variables.

Another clinically relevant observation was the potential prognostic signal of tumor δ^13^C. Patients who died or experienced a DFS event had less negative tumor δ^13^C values, and Cox regression showed borderline associations with both OS and DFS. Although these findings did not reach conventional statistical significance in time-to-event models, the consistency of the direction across outcome comparisons suggests that carbon isotope composition may reflect biologically relevant aspects of tumor metabolism. This result should be interpreted as hypothesis-generating, but it supports further investigation of tumor δ^13^C as a tissue-derived potential prognostic biomarker in OSCC.

Contrary to expectations, we did not observe significant associations between inflammatory markers (NLR, PLR, SII, CRP) and key clinicopathological features such as tumor stage, extranodal extension, or vascular and perineural invasion. This finding contrasts with multiple reports suggesting that elevated systemic inflammation correlates with more advanced disease and adverse pathological features in head and neck cancers. For example, a meta-analysis by Yang L et al. demonstrated that elevated NLR is associated with advanced tumor stage and lymph node involvement in head and neck squamous cell carcinoma (HNSCC) [19]. Similarly, Takenaka Y et al. reported a strong association between systemic inflammatory markers and aggressive disease features [20]. The lack of such associations in our cohort may reflect the relatively small sample size, cohort homogeneity, or differences in tumor biology within OSCC. It also suggests that systemic inflammation may not directly mirror classical histopathological aggressiveness, but instead captures a distinct biological dimension. The predominance of advanced-stage tumors may also have reduced clinicopathological variability and contributed to weaker associations between inflammatory markers and conventional pathological features.

A key aspect of this study was the integration of systemic inflammatory markers with IRMS-derived metabolic parameters. While no associations remained significant after correction for multiple testing, exploratory analyses revealed consistent moderate correlations between NLR and tumor–healthy tissue carbon isotope differences (δ^13^C T–HT), particularly in patients with nodal metastases. This observation is biologically plausible. Stable isotope composition reflects underlying metabolic pathways, including shifts in carbon utilization and nitrogen metabolism. Tumor-associated inflammation may influence metabolic reprogramming through cytokine signaling, immune cell infiltration, and altered nutrient availability.

Previous studies have highlighted the role of metabolic reprogramming in OSCC and its interaction with the tumor microenvironment. For instance, Cairns RA et al. described the central role of altered cellular metabolism in cancer progression [21]. Furthermore, Martinez-Outschoorn UE et al. emphasized the metabolic crosstalk between tumor cells and stromal components [22]. Our findings suggest that systemic inflammation may be linked to tumor metabolic divergence rather than to conventional histopathological features. This represents a novel conceptual link that warrants further investigation.

An important consideration in the interpretation of the present findings is the potential for selection bias related to the study design and eligibility criteria. To reduce the influence of non-cancer-related inflammatory activity on blood-derived biomarkers, patients with active systemic inflammatory or autoimmune diseases, chronic corticosteroid or immunosuppressive therapy, active periodontal inflammation, malnutrition, and diabetes mellitus were excluded from the study cohort. While this approach increased internal consistency and reduced heterogeneity in systemic inflammatory status, it may also have limited the representativeness of the analyzed population. In particular, diabetes mellitus is common among patients with oral squamous cell carcinoma and is known to influence systemic inflammation, immune function, metabolic homeostasis, and potentially cancer outcomes. Therefore, exclusion of diabetic patients may have reduced variability in inflammatory markers such as CRP, NLR, PLR, and SII and may partly explain the relatively weak associations observed between systemic inflammatory indices and clinicopathological features.

Similarly, excluding patients with clinically evident inflammatory or autoimmune conditions may have resulted in a cohort characterized by a narrower inflammatory spectrum than that encountered in routine oncologic practice. Consequently, the observed relationships between inflammatory markers, isotope-derived tumor parameters, and survival outcomes should be interpreted within the context of this selected population. The present findings may therefore not be directly generalizable to all OSCC patients, particularly those with substantial metabolic or inflammatory comorbidity burden. Future multicenter studies including broader and more heterogeneous patient populations will be necessary to determine whether the observed prognostic associations remain stable in real-world clinical settings.

The finding of our study is the independent prognostic value of NLR which is consistent with existing literature. In both univariable and multivariable Cox regression models, higher NLR was significantly associated with worse OS and DFS. Importantly, this effect remained significant after adjustment for nodal status, suggesting that NLR captures prognostic information beyond traditional staging.

These results are consistent with a large body of literature demonstrating the prognostic significance of NLR in HNSCC. A meta-analysis by Mascarella MA et al. confirmed that elevated NLR is associated with poorer survival outcomes [23]. Similarly, Howard R et al. showed that systemic inflammatory markers are independent predictors of survival in OSCC [24].

Biologically, elevated NLR reflects a relative increase in neutrophil-mediated tumor-promoting inflammation and a decrease in lymphocyte-mediated anti-tumor immunity. This imbalance is thought to facilitate tumor progression, angiogenesis, and immune evasion.

Tumor δ^13^C also emerged as a potentially relevant tissue-derived prognostic marker. Patients who died during follow-up or experienced a DFS event had less negative tumor δ^13^C values, and Cox regression showed borderline associations of tumor δ^13^C with both OS and DFS. Although these associations require validation, their consistency suggests that carbon isotope composition may reflect metabolic features linked to tumor aggressiveness. This observation is particularly relevant because δ^13^C represents a local tissue-derived biomarker, in contrast to NLR, which reflects systemic host inflammatory status. The combination of these two domains may therefore provide a broader biological representation of OSCC behavior.

In extended multivariable models, the inclusion of SII alongside NLR resulted in high multicollinearity, as reflected by elevated variance inflation factors. This finding is expected, given that SII incorporates neutrophil and lymphocyte counts and is therefore mathematically related to NLR.

As a result, the primary model including NLR and nodal status provides a more stable and interpretable framework. Extended models should be interpreted with caution and considered exploratory.

Our findings suggest that systemic inflammatory markers, particularly NLR, may serve as accessible and cost-effective potential prognostic biomarkers in OSCC. More importantly, the observed association between inflammation and metabolic tumor characteristics points toward a potential integration of systemic and metabolic profiling.

This combined approach could improve risk stratification and provide insights into tumor biology that are not captured by conventional histopathological assessment.

### Limitations

This study has several limitations. First, the relatively small sample size limits statistical power and may have contributed to the lack of significant findings after multiple testing correction. The cohort size and substantial number of exploratory analyses increase the risk of false-positive findings and model overfitting, particularly in survival analyses. Therefore, all prognostic findings should be interpreted cautiously and considered hypothesis-generating until externally validated. In addition, the analyzed group consisted predominantly of patients with advanced-stage disease (78% stage IV), treated at a tertiary referral center. This limits the generalizability of the findings to broader OSCC populations, particularly patients with early-stage tumors. Detailed quantitative data regarding smoking exposure and alcohol consumption were not consistently available for all patients and therefore were not included in the formal analyses. Residual confounding related to these factors cannot be excluded. Second, the retrospective design introduces potential selection bias. Third, IRMS analysis, while highly informative, is not yet widely available in clinical practice.

Another important limitation is the possibility of residual confounding. Although multivariable models included pathologic nodal status, several clinically relevant factors could not be comprehensively controlled for because of the limited sample size and retrospective design. In particular, detailed quantitative information regarding smoking exposure and alcohol consumption was not consistently available across the cohort. Both factors are strongly associated with systemic inflammatory activation, metabolic alterations, nutritional status, and prognosis in head and neck cancer, and may therefore have influenced both blood-based inflammatory markers and tissue isotope composition.

Residual confounding may also be related to tumor site heterogeneity, treatment-related factors, and coexisting comorbidities. Different oral cavity subsites may exhibit distinct biological behavior, metabolic characteristics, and lymphatic drainage patterns, potentially influencing both inflammatory and survival outcomes. In addition, although all patients underwent guideline-based management, variability in adjuvant treatment modalities and treatment intensity could have affected long-term oncologic outcomes. Furthermore, comorbid conditions not captured by the exclusion criteria may still have contributed to systemic inflammatory status. Given the exploratory nature of the study and the relatively small cohort size, the multivariable models should therefore be interpreted cautiously and regarded primarily as hypothesis-generating rather than definitive evidence of independent causal relationships.

Finally, the exploratory nature of some analyses, particularly subgroup analyses and multiple comparisons, necessitates cautious interpretation of the results.

## 5. Conclusions

This study demonstrates a reproducible tumor-specific isotope and elemental phenotype in oral squamous cell carcinoma. Compared with matched healthy oral mucosa, tumor tissue showed higher nitrogen content, lower carbon content, an increased [N]/[C] ratio, lower δ^15^N, and less negative δ^13^C, indicating that IRMS-derived parameters capture distinct biochemical features of OSCC tissue. Preoperative systemic inflammatory markers, including NLR, PLR, SII, and CRP, were not robustly associated with conventional clinicopathological indicators of tumor aggressiveness after correction for multiple testing. Although WBC and lymphocyte counts were nominally higher in patients with pathologic nodal metastases, these associations did not remain significant after FDR adjustment and should therefore be interpreted as exploratory. Correlations between inflammatory indices and isotope-derived tissue parameters were modest, suggesting that systemic inflammation and local tumor isotope signatures reflect partly distinct biological domains. In survival analyses, higher NLR was associated with poorer overall and disease-free survival, whereas less negative tumor δ^13^C showed a potential adverse prognostic signal. These findings support the complementary value of systemic inflammatory markers and IRMS-based tissue profiling in OSCC, but external validation in a larger, independent cohort is required before clinical implementation.

## Figures and Tables

**Table 1:** Clinicopathological characteristics of the study group.

	Overall Cohort (N = 50)
Age, years, median (IQR)	68 (60.25–71)
Gender, n (%)	
Male	32 (64.0%)
Female	18 (36.0%)
Anatomical site, n (%)	
Lower gingiva	20 (40.0%)
Floor of the mouth	17 (34.0%)
Tongue	10 (20.0%)
Buccal Mucosa	3 (6.0%)
pT category, AJCC 8th, n (%)	
T1	2 (4.0%)
T2	10 (20.0%)
T3	14 (28.0%)
T4	24 (48.0%)
pN category, AJCC 8th, n (%)	
pN0	21 (42.0%)
pN1	2 (4.0%)
pN2B	2 (4.0%)
pN2C	1 (2.0%)
pN3B	24 (48.0%)
pN category, binary, n (%)	
pN0	21 (42.0%)
pN+	29 (58.0%)
Clinical Stage, AJCC 8th, n (%)	
I	2 (4.0%)
II	4 (8.0%)
III	5 (10.0%)
IV	39 (78.0%)
DOI (Depth of invasion), n (%)	
1–5	2 (4.0%)
5.1–10	21 (42.0%)
>10	27 (54.0%)
Tumor Grade n (%)	
G1	9 (18.0%)
G2	31 (62.0%)
G3	10 (20.0%)
Adverse pathological features n (%)	
Extranodal extension (ENE)	23 (46.0%)
Angioinvasion	18 (36.0%)
Neuroinvasion	16 (32.0%)
Oncologic outcomes	
locoregional recurrence	17 (34.0%)
distant metastases	10 (20.0%)
deaths	31 (62.0%)
DFS event: recurrence or death	
Yes	37 (74.0%)
No	13 (26.0%)
DFS time, weeks, median (IQR)	181.5 (71–285.25)
Death/OS event	
Yes	31 (62.0%)
No	19 (38.0%)
OS time, weeks, median (IQR)	208 (157–309.5)

**Table 2:** Inflammatory markers.

Inflammatory Markers	Overall Cohort (N = 50)
NLR, median (IQR)	3.11 (2.39–4.75)
PLR, median (IQR)	151.04 (108.14–190.44)
SII, median (IQR)	704.62 (530.42–1317.19)
Preoperative CRP, mg/L, median (IQR)	6.55 (2.25–15.35)
CRP > 5 mg/L	30 (60.0%)
CRP ≤ 5 mg/L	20 (40.0%)

**Table 3:** Matched tissue IRMS comparisons.

Parameter	Comparison	Sample A, Median (IQR)	Sample B, Median (IQR)	Paired Difference, Median (IQR)	*p*	q (FDR)
x(N)	T vs. M	12.65 (12.04–12.89)	10.48 (9.59–11.87)	1.47 (0.14–2.61)	<0.001	<0.001
x(N)	T vs. HT	12.65 (12.04–12.89)	9.19 (7.65–10.71)	3.19 (1.64–4.22)	<0.001	<0.001
x(N)	M vs. HT	10.48 (9.59–11.87)	9.19 (7.65–10.71)	1.01 (0.11–3.04)	<0.001	<0.001
x(C)	T vs. M	46.21 (45.39–47.13)	50.97 (47.38–53.44)	−3.54 (−6.02–−0.73)	<0.001	<0.001
x(C)	T vs. HT	46.21 (45.39–47.13)	54.13 (50.67–57.92)	−6.49 (−10.83–−2.66)	<0.001	<0.001
x(C)	M vs. HT	50.97 (47.38–53.44)	54.13 (50.67–57.92)	−2.54 (−6.27–−0.12)	<0.001	<0.001
[N]/[C]	T vs. M	0.27 (0.26–0.28)	0.21 (0.17–0.25)	0.05 (0.00–0.09)	<0.001	<0.001
[N]/[C]	T vs. HT	0.27 (0.26–0.28)	0.17 (0.14–0.21)	0.09 (0.04–0.12)	<0.001	<0.001
[N]/[C]	M vs. HT	0.21 (0.17–0.25)	0.17 (0.14–0.21)	0.02 (−0.01–0.08)	<0.001	<0.001
δ^15^N	T vs. M	8.70 (8.31–9.33)	9.62 (9.25–9.96)	−0.77 (−1.37–−0.31)	<0.001	<0.001
δ^15^N	T vs. HT	8.70 (8.31–9.33)	9.81 (9.45–10.27)	−1.04 (−1.52–−0.52)	<0.001	<0.001
δ^15^N	M vs. HT	9.62 (9.25–9.96)	9.81 (9.45–10.27)	−0.26 (−0.64–0.20)	0.047	0.051
δ^13^C	T vs. M	−22.41 (−23.00–−22.05)	−23.34 (−24.27–−22.51)	0.58 (−0.08–1.44)	<0.001	<0.001
δ^13^C	T vs. HT	−22.41 (−23.00–−22.05)	−23.72 (−24.48–−23.25)	1.16 (−0.03–1.84)	<0.001	<0.001
δ^13^C	M vs. HT	−23.34 (−24.27–−22.51)	−23.72 (−24.48–−23.25)	0.20 (−0.37–0.97)	0.103	0.103

Note: Wilcoxon signed-rank test for paired comparisons. FDR correction by Benjamini–Hochberg across paired tissue comparisons.

**Table 4:** Associations between preoperative inflammatory markers and clinicopathological features.

Clinicopathological Feature	Blood Marker: NLR		Mann–Whitney U	*p* Value	FDR q Value
pT	pT1–T2	pT3–T4	147.000	0.067	0.468
	2.71 [1.90–3.36]	3.40 [2.65–4.83]			
pN	pN0	pN+	350.000	0.376	0.728
	3.50 [2.79–4.30]	2.85 [2.21–4.84]			
Stage	I–II	III–IV	136.000	0.919	1.000
	3.07 [2.78–5.10]	3.11 [2.33–4.66]			
DOI	<10 mm	≥10 mm	231.000	0.124	0.634
	2.78 [2.28–3.77]	3.50 [2.76–5.10]			
ENE	No	Yes	352.000	0.425	0.736
	3.36 [2.73–4.45]	2.85 [2.15–5.08]			
Angioinvasion	No	Yes	264.000	0.635	0.880
	3.21 [2.32–4.80]	3.11 [2.68–4.59]			
Neuroinvasion	No	Yes	321.000	0.313	0.709
	3.19 [2.64–5.24]	2.91 [2.05–4.30]			
	**Blood marker: CRP**				
pT	pT1–T2	pT3–T4	165.000	0.156	0.647
	3.60 [1.58–7.72]	7.50 [2.98–16.90]			
pN	pN0	pN+	286.000	0.723	0.929
	5.30 [2.90–9.90]	7.29 [1.80–15.40]			
Stage	I–II	III–IV	104.000	0.411	0.728
	4.70 [2.15–8.75]	7.10 [2.55–15.90]			
DOI	<10 mm	≥10 mm	308.500	0.977	1.000
	5.30 [2.45–22.55]	7.70 [2.40–12.75]			
ENE	No	Yes	334.000	0.654	0.884
	6.10 [3.05–16.00]	7.00 [1.70–12.85]			
Angioinvasion	No	Yes	251.500	0.467	0.758
	5.60 [2.55–11.28]	8.75 [2.08–21.12]			
Neuroinvasion	No	Yes	199.500	0.134	0.634
	5.30 [1.88–9.88]	10.05 [3.85–23.12]			
	**Blood marker: PLR**				
pT	pT1–T2	pT3–T4	200.000	0.532	0.774
	137.50 [84.56–187.84]	159.28 [111.54–190.44]			
pN	pN0	pN+	347.000	0.409	0.728
	153.37 [129.22–190.91]	148.57 [93.30–185.14]			
Stage	I–II	III–IV	131.000	0.988	1.000
	150.63 [100.65–185.32]	152.54 [109.43–189.50]			
DOI	<10 mm	≥10 mm	278.000	0.533	0.774
	147.88 [100.40–187.08]	162.18 [126.15–193.30]			
ENE	No	Yes	326.000	0.770	0.946
	153.37 [113.01–189.97]	148.57 [98.45–189.10]			
Angioinvasion	No	Yes	342.000	0.280	0.709
	159.36 [126.05–191.45]	134.66 [95.87–177.12]			
Neuroinvasion	No	Yes	325.000	0.275	0.709
	155.53 [123.92–190.44]	138.23 [91.92–171.43]			
	**Blood marker: SII**				
pT	pT1–T2	pT3–T4	151.000	0.082	0.503
	636.94 [342.52–861.56]	733.02 [562.14–1419.60]			
pN	pN0	pN+	304.000	1.000	1.000
	696.60 [587.77–1129.93]	712.64 [500.92–1400.80]			
Stage	I–II	III–IV	121.000	0.760	0.946
	759.60 [451.52–1244.45]	704.62 [533.77–1197.64]			
DOI	<10 mm	≥10 mm	252.000	0.259	0.709
	680.24 [431.93–979.19]	739.18 [544.52–1455.68]			
ENE	No	Yes	318.000	0.892	1.000
	726.86 [558.26–1254.77]	687.89 [518.18–1235.75]			
Angioinvasion	No	Yes	280.000	0.880	1.000
	719.75 [547.38–1085.51]	692.24 [509.55–1419.60]			
Neuroinvasion	No	Yes	334.000	0.201	0.694
	733.02 [598.04–1399.77]	594.84 [496.75–864.88]			

Values are medians [IQR]. *p*-values from Mann–Whitney U test; q-values from Benjamini–Hochberg FDR correction.

**Table 5:** Selected blood and isotope-derived biomarkers according to pathologic nodal status.

Variable	Overall, Median (IQR)	pN0, Median (IQR)	pN+, Median (IQR)	*p*	q (FDR)	Rank-Biserial r
WBC	7.75 (6.53–10.10)	7.40 (6.10–8.00)	9.50 (7.10–10.60)	0.021	0.520	0.39
NEU	5.45 (4.29–7.04)	4.73 (4.12–6.36)	5.66 (4.67–7.46)	0.103	0.815	0.27
LYM	1.65 (1.32–1.94)	1.55 (1.13–1.65)	1.88 (1.40–2.53)	0.031	0.520	0.36
NLR	3.11 (2.39–4.75)	3.50 (2.79–4.30)	2.85 (2.21–4.84)	0.376	0.815	−0.15
PLR	151.04 (108.14–190.44)	153.37 (129.22–190.91)	148.57 (93.30–185.14)	0.409	0.815	−0.14
SII	704.62 (530.42–1317.19)	696.60 (587.77–1129.93)	712.64 (500.92–1400.80)	1.000	1.000	0.00
CRP before surgery	6.55 (2.25–15.35)	5.30 (2.90–9.90)	7.29 (1.80–15.40)	0.723	1.000	0.06
Tumor nitrogen	12.65 (12.04–12.89)	12.49 (11.77–12.70)	12.72 (12.30–12.95)	0.065	0.733	0.31
Tumor δ^13^C	−22.41 (−23.00–−22.05)	−22.52 (−23.01–−22.29)	−22.35 (−22.99–−21.84)	0.335	0.815	0.16
ΔT–M δ^13^C	0.58 (−0.08–1.44)	0.33 (0.07–0.71)	1.07 (−0.13–1.64)	0.195	0.815	0.22
ΔT–HT δ^13^C	1.16 (−0.03–1.84)	0.98 (−0.30–1.67)	1.25 (0.06–1.88)	0.542	0.921	0.10

Note: Mann–Whitney U test for pN0 vs. pN+. Rank-biserial r estimates direction and magnitude; positive values indicate higher values in pN+ patients.

**Table 6:** Univariable Cox proportional hazards models for OS and DFS.

Endpoint	Variable	Scale	HR (95% CI)	*p*
OS	NLR	per IQR increase	1.14 (1.01–1.30)	0.041
OS	pN+	binary	2.21 (1.03–4.72)	0.041
OS	Tumor δ^13^C	per IQR increase	1.34 (0.95–1.88)	0.092
OS	SII	per IQR increase	1.16 (0.98–1.37)	0.094
OS	ENE	binary	1.77 (0.87–3.62)	0.117
OS	CRP before surgery	per IQR increase	1.03 (0.94–1.12)	0.556
DFS	NLR	per IQR increase	1.16 (1.00–1.33)	0.047
DFS	Tumor δ^13^C	per IQR increase	1.34 (0.98–1.83)	0.066
DFS	SII	per IQR increase	1.18 (0.98–1.43)	0.076
DFS	CRP before surgery	per IQR increase	1.09 (0.98–1.22)	0.114
DFS	pN+	binary	1.76 (0.87–3.57)	0.117
DFS	ENE	binary	1.58 (0.80–3.12)	0.189

Note: Continuous biomarkers were scaled per interquartile range increase. Binary variables are shown as present vs. absent. Given the sample size and event number, these models are univariable and exploratory.

## Data Availability

The data on which this study is based will be made available upon request at https://www.researchgate.net/profile/Katarzyna-Bogusiak (accessed on 28 April 2026).

## Supplementary Materials

The following supporting information can be downloaded at: https://www.mdpi.com/article/10.3390/jcm15135278/s1, Figure S1. Collinearity diagnostics.

## Appendix A

### STROBE Checklist for Observational Studies

Manuscript title: Systemic Inflammation, Tumor Isotopic Signatures, and Prognosis in Oral Squamous Cell Carcinoma: Exploratory Integration of Blood- and Tissue-Derived Biomarkers—An Exploratory Retrospective Secondary Analysis

Study design: Retrospective secondary analysis of prospectively collected data.

Reporting guideline: The manuscript was prepared in accordance with the STROBE (Strengthening the Reporting of Observational Studies in Epidemiology) statement.

**Table A1:** STROBE checklist for observational studies and corresponding locations of the relevant reporting items in the manuscript.

Section/Item.	Recommendation	Reported on Page(s)
**Title and abstract**		
1(a)	Indicate the study design in the title or abstract	Title page; Abstract
1(b)	Provide an informative and balanced summary	Abstract
**Introduction**		
2	Explain the scientific background and rationale	Introduction
3	State-specific objectives and hypotheses	End of Introduction
**Methods**		
4	Present key elements of the study design early in the paper	Section 2.1
5	Describe the setting, locations, and relevant dates	Section 2.1
6(a)	Describe eligibility criteria and participant selection	Section 2.2
7	Clearly define outcomes, exposures, predictors, confounders	Section 2.2, Section 2.3, Section 2.4 and Section 2.5
8	Describe data sources and methods of assessment	Section 2.3, Section 2.4 and Section 2.5
9	Describe efforts to address potential bias	Section 2.2 and Section 2.6 and Section Limitations
10	Explain how the study size was determined	Section Limitations
11	Explain the handling of quantitative variables	Section 2.3 and Section 2.6
12(a)	Describe all statistical methods	Section 2.6
12(b)	Describe methods used to examine subgroups/interactions	Section 2.6, Section 3.5 and Section 3.6
12(c)	Describe analytical methods considering the sampling strategy	Section 2.6
12(d)	Describe sensitivity analyses	Section 3.8
**Results**		
13(a)	Report participant numbers at each stage	Section 3.1
13(b)	Give reasons for non-participation/exclusion	Section 2.2
13(c)	Consider the use of a flow diagram	Not included
14(a)	Give characteristics of study participants	Table 1
14(b)	Indicate the number of participants with missing data	Section 2.2
15	Report outcome events or summary measures	Section 3.1 and Section 3.7
16(a)	Give unadjusted and adjusted estimates	Table 4, Table 5 and Table 6
16(b)	Report category boundaries when continuous variables are categorized	Section 2.3
16(c)	Translate relative risk into absolute risk if relevant	Not applicable
17	Report additional analyses	Section 3.5, Section 3.6, Section 3.7 and Section 3.8
**Discussion**		
18	Summarize key results	Discussion opening paragraph
19	Discuss limitations and potential bias	Section Limitations
20	Provide a cautious overall interpretation	Discussion
21	Discuss generalizability	Section 4 and Section Limitations
**Other information**		
22	Give the source of funding and the role of funders	Funding statement
