# Supplementary figures and images for: Systemic Inflammation, Tumor Isotopic Signatures, and Prognosis in Oral Squamous Cell Carcinoma: Exploratory Integration of Blood- and Tissue-Derived Biomarkers—An Exploratory Retrospective Secondary Analysis
Source: J Clin Med. 2026 Jul 6;15(13):5278. doi: 10.3390/jcm15135278 (PMC13363046; doi:10.3390/jcm15135278)

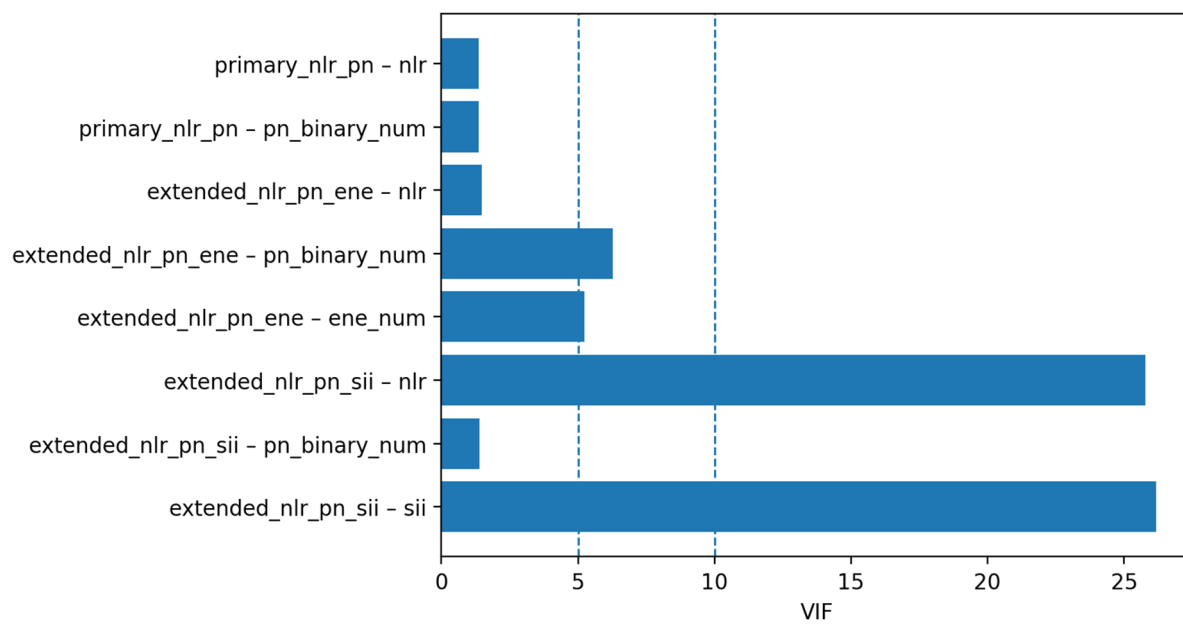

**Figure S1.** Collinearity diagnostics.

Supplement: Supplementary file 1 [file jcm-15-05278-s001.zip › jcm-4351041-supplementary.pdf]
